# Fertility and Pregnancy-Associated ß-Cell Proliferation in Mice Deficient in Proglucagon-Derived Peptides

**DOI:** 10.1371/journal.pone.0043745

**Published:** 2012-08-23

**Authors:** Chisato Sugiyama, Michiyo Yamamoto, Tomomi Kotani, Fumitaka Kikkawa, Yoshiharu Murata, Yoshitaka Hayashi

**Affiliations:** 1 Department of Genetics, Research Institute of Environmental Medicine, Nagoya University, Nagoya, Japan; 2 Department of Obstetrics and Gynecology, Nagoya University Graduate School of Medicine, Nagoya, Japan; University of Ulster, United Kingdom

## Abstract

Proglucagon, which is encoded by the glucagon gene (*Gcg*), is the precursor of several peptide hormones, including glucagon and glucagon-like peptide 1 (GLP-1). Whereas glucagon stimulates hepatic glycogenolysis and gluconeogenesis, GLP-1 stimulates insulin secretion to lower blood glucose and also supports ß-cell proliferation and protection from apoptotic stimuli. Pregnancy is a strong inducer of change in islet function, however the roles of proglucagon-derived peptides in pregnancy are only partially understood. In the present study, we analyzed fertility and pregnancy-associated changes in homozygous glucagon-green fluorescent protein (gfp) knock-in mice (*Gcg^gfp/gfp^*), which lack all the peptides derived from proglucagon. Female *Gcg^gfp/gfp^* mice could deliver and raise *Gcg^gfp/gfp^* pups to weaning and *Gcg^gfp/gfp^* pups from *Gcg^gfp/gfp^* dams were viable and fertile. Pregnancy induced ß-cell proliferation in *Gcg^gfp/gfp^* mice as well as in control mice. However, serum insulin levels in pregnant *Gcg^gfp/gfp^* females were lower than those in control pregnant females under ad libitum feeding, and blood glucose levels in pregnant *Gcg^gfp/gfp^* females were higher after gestational day 12. *Gcg^gfp/gfp^* females showed a decreased pregnancy rate and smaller litter size. The rate of successful breeding was significantly lower in *Gcg^gfp/gfp^* females and was not improved by experience of breeding. Taken together, proglucagon-derived peptides are not required for pregnancy-associated ß-cell proliferation, however, are required for regulation of blood glucose levels and normal reproductive capacity. *Gcg^gfp/gfp^* mice may serve as a novel model to analyze the effect of mild hyperglycemia during late gestational periods.

## Introduction

Pregnancy is one of the strongest physiological stimuli that induce structural and functional changes in pancreatic islet ß-cells. Both ß-cell mass and insulin secretion are increased and the threshold for glucose-stimulated insulin secretion is decreased during pregnancy [1], [2], [3], [4], and insufficiency in such responses can lead to the development of gestational diabetes mellitus (GDM) [5], [6], [7]. Therefore, pathogenesis of type 2 diabetes mellitus (DM) and that of GDM overlaps, and the women who have a history of GDM are under high risk of developing type 2 DM in later life [7], [8].

The molecular mechanisms that are involved in altered ß-cell function during pregnancy are not fully understood. Previous studies have shown that prolactin (PRL), placental lactogens (PLs), and serotonin play important roles in the pregnancy-associated changes in ß-cell mass and function in rodents [4], [9], [10], [11]. On the other hand, involvement of glucagon-like peptide-1 (GLP-1) in the pregnancy-associated changes in ß-cell mass and function has not been investigated, although it is well established that GLP-1 supports β-cell proliferation and inhibits apoptosis of these cells [12], [13], [14], [15], [16].

Tissue-specific posttranslational processing of proglucagon produces multiple peptides that harbor apparently counteracting functions. Glucagon is produced in pancreatic islet α-cells through the cleavage of proglucagon by prohormone convertase 2 (Pcsk2) and it stimulates hepatic glycogenolysis and gluconeogenesis to increase blood glucose levels. GLP-1 is produced in intestinal L-cells through the cleavage of proglucagon by prohormone convertase 1/3 (Pcsk1) and is released in response to nutrient ingestion. Compared with glucagon and GLP-1, the physiological roles of other proglucagon-derived peptides including GLP-2, glicentin and oxyntomodulin are far less understood.

We have recently established a mouse model, in which the glucagon gene is disrupted by introduction of the cDNA for green fluorescentr protein (GFP; *Gcg^gfp/+^*). Homozygous *Gcg^gfp/gfp^* mice are born in the expected Mendelian ratio and appear grossly normal, despite the absence of proglucagon-derived peptides including glucagon and GLP-1 [16]. Interestingly, the blood glucose levels in adult *Gcg^gfp/gfp^* mice are not significantly different from those in the control mice. Therefore, the *Gcg^gfp/gfp^* mice make contrasts with two animal models defective in glucagon production or action, *Pcsk2^−/−^* and glucagon receptor-deficient (*Gcgr^−/−^*) mice, both of which display lower blood glucose levels and markedly elevated serum GLP-1 concentrations.

In the present study, we analyzed the fertility of *Gcg^gfp/gfp^* mice to clarify the roles of proglucagon-derived peptides in pregnancy and pregnancy-associated changes in ß-cell mass and function.

## Materials and Methods

### Animals and Experimental Set up

Heterozygous Gcg-EGFP knock-in mice (*Gcg^gfp/+^*) were generated as described previously [16]. In brief, the murine glucagon gene was disrupted by replacing the region of the glucagon gene that spans from exon 2 to exon 5 with GFP cDNA, a polyadenylation signal, and an expression cassette for neomycin resistance. *Gcg^gfp/+^* mice were backcrossed into the C57/BL6J background for more than 10 generations. *Gcg^gfp/+^* female mice were bred with *Gcg^gfp/+^* males to generate homozygous (*Gcg^gfp/gfp^*) offspring. All mice were housed in specific pathogen-free barrier facilities at the Research Institute of Environmental Medicine, Nagoya University, and maintained on a 12-h light, 12-h dark cycle and constant temperature (23°C) with free access to certified chow (Lab Animal Diet MF; Oriental Yeast Co. Ltd., Tokyo, Japan) and distilled water. All procedures were performed in accordance with a protocol approved by the Nagoya University Institutional Animal Care and Use Committee. To obtain pregnant females, female mice aged 3–4 months were housed with male mice in all combinations of genotypes. The presence of a mucous vaginal plug indicated that mating had occurred during the previous night, and the time point was designated as gestational day (G) 0.5 ( = embryonic day (E) 0.5).

### Blood Glucose and Serum Insulin Levels

Blood samples were obtained via tail bleeding and from severed neck vessels in adult female mice and neonates respectively. Fasting blood samples were collected following overnight fasting (16–18 hours). Glucose levels were determined using a Medisafe glucose meter (TERUMO, Tokyo, Japan). Serum insulin levels ware measured in duplicate using the High Sensitivity PLUS insulin kit (Morinaga-Seikagaku Co. Ltd., Yokohama, Japan) in accordance with the manufacturer’s instructions.

### Oral Glucose Tolerance Test (OGTT)

After a 16-h period of fasting, a glucose load of 2 g/kg was administered to conscious mice orally. Blood samples were collected at 0, 15, 30, 60, 90, and 120 minutes after the administration of glucose.

### Pancreatic Insulin Content

Mice were sacrificed by cervical dislocation and the entire pancreas was dissected free from other tissue immediately. Following the measurement of the weight, the pancreas was homogenized in 8 ml of acid ethanol (70% ethanol, 1.5%HCl). After overnight incubation at −20°C, the suspension was centrifuged at 2000 rpm for 15 minutes at 4°C. The supernatant was collected and neutralized. The extract was analyzed for insulin content [17].

### Immunohistochemistry and Morphometry

Adult female mice were perfused intracardially with 4% paraformaldehyde (PFA) in phosphate-buffered saline (PBS) and post-fixed for 4 hours in the same fixative. Embryos and pups were killed by decapitation, and the pancreases were dissected and fixed for 4 hours in the same fixative saline. Fixed tissues were cryoprotected with graded sucrose (10∼30%) in PBS followed by immersion into OCT compound (Sakura Co. Ltd., Tokyo, Japan) and were then frozen in liquid N_2_. Twenty micrometer sections were prepared using a Microm HM500 OM Microtome Cryostat (Carl Zeiss Japan, Tokyo, Japan).

The following primary antibodies were used: guinea pig polyclonal anti-insulin antibody (diluted to 1∶300; Abcam plc., Cambridge, UK), rabbit polyclonal anti-glucagon antibody (1∶200; Abcam, plc., Cambridge, UK), and rabbit polyclonal anti-serotonin antibody (1∶500; Incstar, Stillwater, Minn., USA). After incubation with primary antibodies at 4°C overnight, the sections were incubated with Cy3-, Alexa 568-, or Alexa 633-labeled species-specific anti-IgG antibodies (diluted to 1∶250∼500; Life Technologies Japan, Tokyo, Japan) for 1 h. The immunostained sections were examined with a Zeiss LSM 710 confocal laser-scanning microscope (Carl Zeiss Microscopy, Tokyo, Japan).

Quantitative analyses of ß-cell mass were performed on three to four sections of each pancreas at 200-µm intervals. Each section was scanned using a NanoZoomer 2.0 RS whole slide scanner (Hamamatsu Photonics, Hamamatsu City, Japan) to generate high resolution images at 20× magnification. Images were analyzed with Image-Pro Plus 6.1 software (Media Cybernetics, Silver Springs, MD) to measure the areas of insulin-positive cells as well as the total area of pancreatic sections. The ß-cell mass was calculated by multiplying the weight of pancreas by the average percentage area of insulin-positive cells relative to the total area of pancreatic sections. Results represent the average of three animals from each genotype.

### Statistical Analysis

The quantitative data are presented as the means±SEM. The statistical significance of the differences between two groups was determined by Student’s *t*-test. Differences among more than two groups were calculated with one-way ANOVA followed by Turkey’s test, using IBM SPSS Statistics software Version 19. The deviation of a given genotype from the expected Mendelian ratio and fertility parameters were analyzed by the chi-square test using the same software. p<0.05 was considered to be statistically significant.

## Results

We first analyzed the effect of parental *Gcg* genotype on pregnancy and breeding parameters. Female mice were mated with male mice in all combinations of genotypes. Given that no significant differences in fertility, blood glucose levels and body weights were observed between *Gcg^gfp/+^* and *Gcg^+/+^* females (data not shown), we compared the data for *Gcg^gfp/gfp^* mice with the combined data for *Gcg^+/+^* and *Gcg^gfp/+^* mice as the control. Female mice were considered to be infertile if the presence of vaginal plugs was confirmed twice or more in a time interval of more than 4 weeks, and no subsequent successful pregnancy occurred. The proportion of infertile *Gcg^gfp/gfp^* females was not significantly different from that of the infertile control females (7.4 percent vs. 4.4 percent, *P* = 0.48). The genotype of the male mice did not affect the pregnancy outcomes as indicated in Table 1.

**Table 1:** Genotype of offspring from multiple matings analyzed at P0.

Genotype of pairs					Offspring				*p*
			No. of pairs	Mean litter size^a^			genotype ratio		
female		male			genotype	n	observed	(expected)	
*+/+*	x	*gfp/+*	10	6.5±0.7	*+/+*	30	0.46	(0.5)	0.54
					*gfp/+*	35	0.54	(0.5)	
*+/+*	x	*gfp/gfp*	9	6.6±0.9	*gfp/+*	59	–		–
*gfp/+*	x	*gfp/+*	20	7.4±0.5	*+/+*	43	0.29	(0.25)	0.43
					*gfp/+*	72	0.49	(0.5)	
					*gfp/gfp*	32	0.22	(0.25)	
*gfp/+*	x	*gfp/gfp*	9	7.0±0.8	*gfp/+*	36	0.57	(0.5)	0.26
					*gfp/gfp*	27	0.43	(0.5)	
*gfp/gfp*	x	*gfp/+*	16	3.8±0.6	*gfp/+*	30	0.49	(0.5)	0.90
					*gfp/gfp*	31	0.51	(0.5)	
*gfp/gfp*	x	*gfp/gfp*	9	3.9±0.6	*gfp/gfp*	35	–		–

Data were compared by the chi-square test.

a Values are expressed as means±SEM.

**Figure 1:**
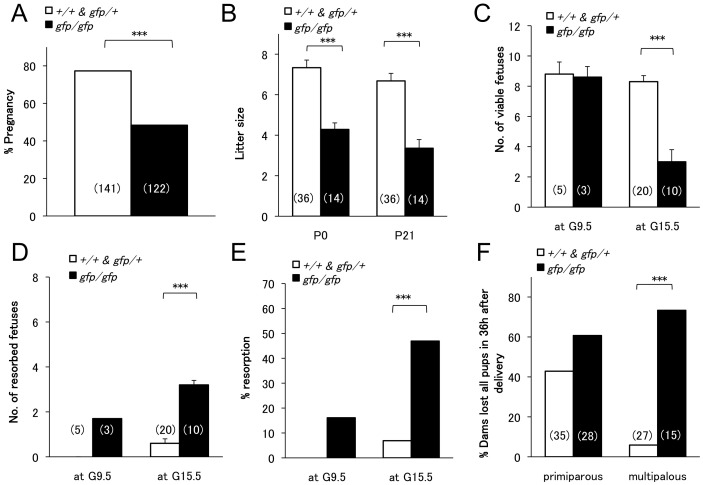
Fertility, litter size and survival of fetuses/pups in *Gcg^gfp/gfp^* mice. (A) Pregnancy rate in the control (open squares: *Gcg^+/+^* and *Gcg^gfp/+^*) and *Gcg^gfp/gfp^* (closed squares) mice. Numbers in brackets indicate mating. The rate was not affected by genotype of the male (data not shown). ***p<0.01 by the chi-square test. (B) Litter size in the control and *Gcg^gfp/gfp^* (closed squares) mice at postnatal day 0 and 21. Numbers in brackets indicate litters analyzed. Values are expressed as means±SEM. ***p<0.01 by one-way ANOVA and t-test. (C–E) Survival of fetuses. Female mice were sacrificed at gestational day 9.5 and 15.5. Numbers in brackets indicate number of pregnant females analyzed. Numbers of viable fetuses (C), resorbed fetuses (D) and % resorption (E) are shown. Values are expressed as means±SEM. ***p<0.01 by one-way ANOVA and t-test. (F) Rate of losing litters within 36 hours after delivery. Values are expressed as means±SEM. ***p<0.01 by one-way ANOVA and t-test.

The pregnancy rate of *Gcg^gfp/gfp^* females was significantly lower than that of the control females (Fig. 1A), but most of pregnant *Gcg^gfp/gfp^* females could deliver at term. As *Gcg^gfp/gfp^* females produced smaller litters than the control females (Fig. 1B), we sacrificed pregnant females at G9.5 or G15.5 to determine the gestational age at which emryos were lost. Although the number and survival rate of embyos in *Gcg^gfp/gfp^* dams were not significantly different from those in control dams at G9.5, approximately half of the embryos in *Gcg^gfp/gfp^* dams were resorbed at G15.5 (Fig. 1C–E). These results indicate that maternal proglucagon-derived peptides are required to fully sustain survival of embryos. We then compared the proportion of dams that had lost all pups within 36 hours after birth for both primiparous and multiparous dams. In most cases, postnatal survival of the litter followed an all-or-none pattern regardless of maternal genotype. At primiparas, no significant difference in neonatal survival was observed between the *Gcg^gfp/gfp^* and control dams (Fig. 1F). However, neonatal survival was not improved by prior birthing experience in *Gcg^gfp/gfp^* females, whereas most of the multiparous *Gcg^+/+^* and *Gcg^gfp/+^* dams could raise their pups (Fig. 1F). These results suggest that proglucagon-derived peptides are involved in breeding behavior of the dams.

Although *Gcg^gfp/gfp^* females produced smaller numbers of pups per a litter and lost litters more frequently than the control females as described above, pups from *Gcg^gfp/gfp^* dams that survived the first postnatal 36 hours grew as well as pups from control dams and the genotypes of the pups were in accordance with the expected Mendelian ratio (Table 1). At post partum day 0 (P0), no significant difference in blood glucose levels was observed among pups from *Gcg^+/+^*, *Gcg^gfp/+^* and *Gcg^gfp/gfp^* dams (Table 2). Although the underlying mechanism is obscure, the mean body weight of *Gcg^gfp/gfp^* pups from *Gcg^gfp/gfp^* dams was greater than that of other types of pup (Table 2). *Gcg^gfp/gfp^* pups that were born to *Gcg^gfp/gfp^* dams survived and grew to adulthood and their fertility was not significantly different from that of *Gcg^gfp/gfp^* pups born to *Gcg^gfp/+^* dams (data not shown).

**Table 2:** Body weight and blood glucose levels of neonates at P0.

Genotype of neonates	Dam	N	Body weight (g)	Blood glucose (mM)
*+/+*_&_ *gfp/+*	from *gfp/+*	108	1.37±0.03^a^	1.89±0.09
*gfp/gfp*	from *gfp/+*	21	1.37±0.02^b^	1.76±0.16
*gfp/+*	from *gfp/gfp*	14	1.32±0.04^c^	1.96±0.25
*gfp/gfp*	from *gfp/gfp*	15	1.52±0.03^abc^	2.16±0.24

Data were compared by one-way ANOVA.

Values are expressed as means±SEM.

a,b,c, Data in the same column with the same letter denote a significant difference between groups (*p*<0.01).

**Figure 2:**
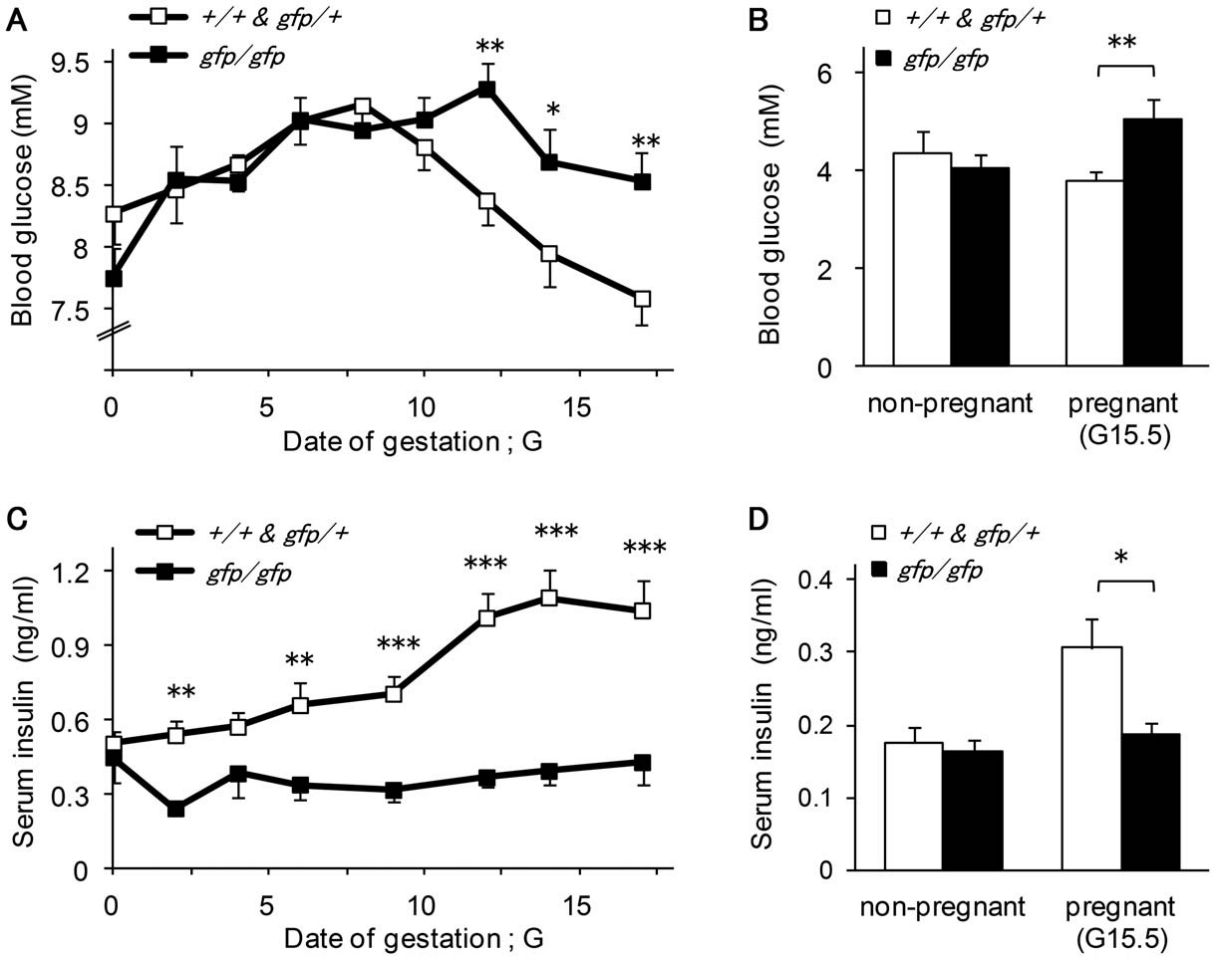
Blood glucose and serum insulin levels in pregnant *Gcg^gfp/gfp^* mice. Blood glucose levels (A) (n = 49–51) and serum insulin levels (C) (n = 8–14) in *ad libitum*-fed *Gcg^gfp/gfp^* mice during pregnancy. Fasting blood glucose levels (B) (n = 9–11) and serum insulin levels (D) (n = 6–14) in non-pregnant *Gcg^gfp/gfp^* and pregnant *Gcg^gfp/gfp^* mice. Open squares: *Gcg^+/+^* and *Gcg^gfp/+^*; closed squares: *Gcg^gfp/gfp^*. Values are expressed as means±SEM. *p<0/05, **p<0.01, ***p<0.001.

We have shown previously that blood glucose levels in *Gcg^gfp/gfp^* females are not significantly different from those in the control females even after 16 hours of fasting [16]. Serum insulin levels in *Gcg^gfp/gfp^* mice are significantly lower than those in control mice under *ad libitum* feeding, and are comparable after 16 hours of fasting [16]. To investigate the role of proglucagon-derived peptides in the regulation of blood glucose levels during pregnancy, blood glucose levels and serum insulin levels were measured in pregnant mice throughout gestation. During early gestation, blood glucose levels under *ad libitum* feeding increased gradually in both *Gcg^gfp/gfp^* and control mice. However, blood glucose levels were significantly higher in *Gcg^gfp/gfp^* mice than in control females during late gestation. In control females, blood glucose levels decreased progressively from mid to late gestation, and the levels at late gestation were lower than those before pregnancy. In contrast, pregnant *Gcg^gfp/gfp^* mice maintained higher blood glucose levels during the period of mid gestation and the levels declined only slightly during late gestation (Figure 2A). Fasting blood glucose levels were also significantly higher in pregnant *Gcg^gfp/gfp^* mice than in pregnant control females at G15.5 (Figure 2B). Serum insulin levels in pregnant *Gcg^gfp/gfp^* mice were maintained at a lower level and no remarkable change was observed throughout gestation. In contrast, in pregnant control females, serum insulin levels increased steadily throughout the duration of pregnancy. Accordingly, the differences in serum insulin levels between *Gcg^gfp/gfp^* and control mice under *ad libitum* feeding became more notable during late gestation (Figure 2C). Fasting serum insulin levels were also significantly lower in *Gcg^gfp/gfp^* mice than in control females at G15.5 (Figure 2D). These results indicate that higher blood glucose levels in pregnant *Gcg^gfp/gfp^* mice are attributable to the impairment of insulin secretion to meet the enhanced metabolic demand that accompanies pregnancy.

**Figure 3:**
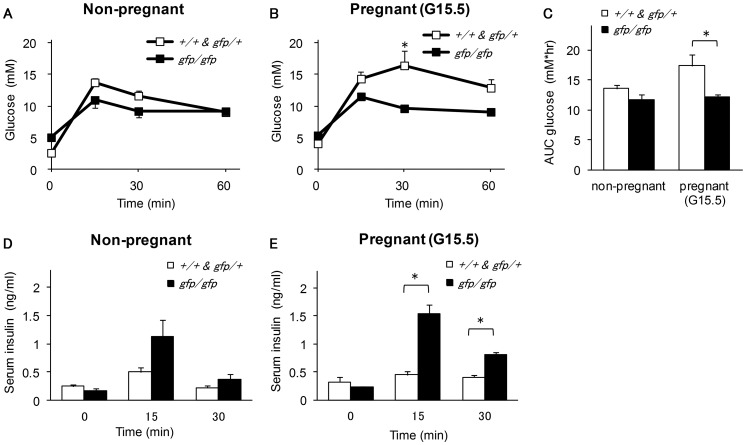
Oral glucose tolerance test (OGTT) in non-pregnant and pregnant *Gcg^gfp/gfp^* mice. Blood glucose levels in non-pregnant *Gcg^gfp/gfp^* mice (A) and pregnant *Gcg^gfp/gfp^* mice (B) and the area under the curve for blood glucose levels(C) during OGTT. Serum insulin levels in non-pregnant *Gcg^gfp/gfp^* mice (D) and pregnant *Gcg^gfp/gfp^* mice (E) from 0 to 30 minutes following OGTT. Open squares: *Gcg^+/+^* and *Gcg^gfp/+^* (n = 3–4); closed squares: *Gcg^gfp/gfp^* (n = 3–5). Values are expressed as means±SEM. *p<0.05.

Given that the higher blood glucose levels and lower serum insulin concentration in pregnant *Gcg^gfp/gfp^* mice suggested the involvement of proglucagon derived-peptides in the alteration of islet function during pregnancy, we performed OGTTs in non-pregnant and pregnant (G15.5) mice. Among the non-pregnant mice, there was no significant difference between *Gcg^gfp/gfp^* and control mice in blood glucose levels and the area under the curve (Figures 3A and 3C). Although the difference did not reach statistical significance, serum insulin levels at 15 min after glucose injection were higher in non-pregnant *Gcg^gfp/gfp^* mice than in control mice (Figure 3B). Despite the presence of higher blood glucose levels and lower insulin levels in pregnant *Gcg^gfp/gfp^* mice under *ad libitum* feeding, these mice showed improved glucose tolerance on the OGTT as compared with pregnant control mice. Blood glucose levels at 30 min after glucose injection and the area under the curve were significantly lower in pregnant *Gcg^gfp/gfp^* mice than in pregnant control mice, and serum insulin levels from 15 to 30 min after glucose injection were significantly higher in the former (Figures 3C–E).

**Figure 4:**
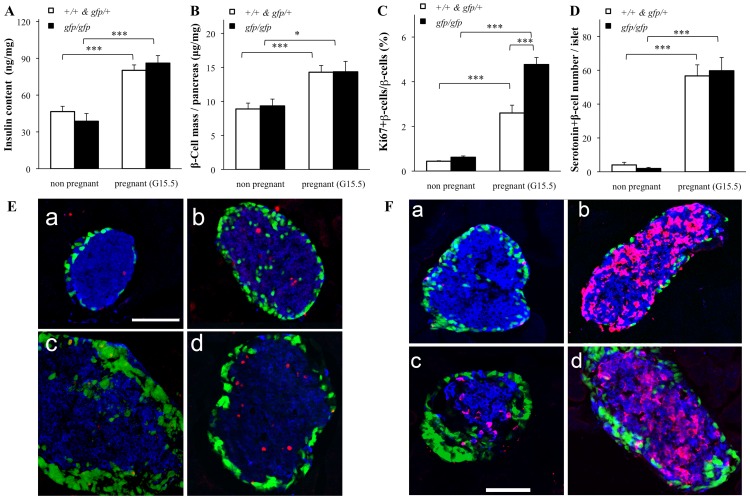
Pregnancy-induced ß-cell adaptations in *Gcg^gfp/gfp^* mice. A, Pancreatic insulin content was measured after a 16-h fasting period (n = 5–7). B, ß-Cell mass was calculated as the relative percentage area of ß-cells (defined as the percentage area of pancreatic sections that stained positive for insulin) multiplied by the weight of the pancreas (n = 3). C, The average number of ki-67-positive ß-cells per ß-cells (n = 3). D, The percentage of serotonin-positive ß-cells per islet (n = 3). Open squares: *Gcg^+/+^* and *Gcg^gfp/+^*; closed squares: *Gcg^gfp/gfp^*. Values are expressed as means±SEM. *p<0.05, **p<0.01, ***p<0.001. E, Immunohistochemical-autofluorescent analyses for GFP (green), insulin (blue) and Ki 67 (red). Islets in non-pregnant *Gcg^gfp/+^* (a), pregnant *Gcg^gfp/gfp^* (b), non-pregnant *Gcg^gfp/+^* (c) and pregnant *Gcg^gfp/gfp^* (d) pancreas. F, Immunohistochemical-autofluorescent analyses for GFP (green), insulin (blue) and serotonin (magenta). Islets in non-pregnant *Gcg^gfp/+^* (a), pregnant *Gcg^gfp/gfp^* (b), non-pregnant *Gcg^gfp/+^* (c) and pregnant *Gcg^gfp/gfp^* (d) pancreas. Scale bars: 100 µm.

To investigate whether ß-cells proliferate during pregnancy in *Gcg^gfp/gfp^* mice, the insulin content of the whole pancreas, ß-cell mass and the percentage of ß-cells positive for the proliferation marker Ki-67 in pancreatic sections were compared. In both in the *Gcg^gfp/gfp^* and control mice, the pancreatic insulin content and the ß-cell mass in the pancreas increased significantly during pregnancy (Figures 4A and 4B) and no significant difference in these parameters was observed between the *Gcg^gfp/gfp^* and control mice regardless of pregnant status. There was no significant difference in the number of Ki-67-positive ß-cells per islet between pregnant *Gcg^gfp/gfp^* and pregnant control mice. However, the percentage of Ki-67-positive ß-cells per islet was significantly higher in pregnant *Gcg^gfp/gfp^* mice than in pregnant control mice because the number of ß-cells per islet was lower in *Gcg^gfp/gfp^* mice than in control mice (Figure 4C and 4E). Therefore, these results indicate that pregnancy-induced ß-cell proliferation in *Gcg^gfp/gfp^* mice is, at least, not attenuated compared to that in control mice.

Recent studies have shown that inhibition of serotonin synthesis in ß-cells leads to the impairment of ß-cell mass expansion and glucose intolerance. Serotonin synthesis is induced by the signaling of PLs and increases during pregnancy [18], [19]. Therefore, we also analyzed the percentage area of ß-cells positive for serotonin in pancreatic sections from pregnant *Gcg^gfp/gfp^* and control mice. The percentage area of serotonin-positive ß-cells was enhanced in both pregnant *Gcg^gfp/gfp^* mice and control mice (Figure 4D and 4F). These results indicated that proglucagon-derived peptides were not required for pregnancy-induced ß-cell proliferation.

## Discussion

In the present study, we observed that *Gcg^gfp/gfp^* mice could deliver and raise *Gcg^gfp/gfp^* pups to weaning despite the absence of proglucagon-derived peptides. This finding contrasted sharply with the results of a previous study in *Gcgr^−/−^* mice, in which *Gcgr^−/−^* pups born to *Gcgr^−/−^* mothers showed delayed maturation of islet cells and died within 24 h after birth [20]. Given that maternal hypoglycemia leads to fetal growth retardation [21], [22], [23], it is likely that the persistently lower blood glucose levels that is observed in *Gcgr^−/−^* mice is responsible for the growth failure of *Gcgr^−/−^* pups born to *Gcgr^−/−^* dams, rather than the absence of glucagon action. In *Gcgr^−/−^* mice, not only are glucagon levels increased markedly, but also GLP-1 levels. The lower blood glucose levels in *Gcgr^−/−^* mice can be attributed to the elevated GLP-1 levels, because blood glucose levels in *Gcgr/GLP-1 receptor* (*Glp1r)* double knockout mice are normal, as are those in *Gcg^gfp/gfp^* mice [24].

In the present study, we also found that ß-cell proliferation was induced by pregnancy in *Gcg^gfp/gfp^* mice as well as in control mice. Therefore, proglucagon-derived peptides are not required for pregnancy-induced ß-cell proliferation. GLP-1 has been shown to increase expression of the Pancreas duodenal homeobox 1 (Pdx-1) gene, thereby stimulating ß-cell proliferation [14]. However, Pdx-1 levels are not increased by PRL [25], which plays important roles in ß-cell proliferation during pregnancy [4], [9], [10], [11]. Several studies indicate that signaling pathways for PRL and those for GLP-1 are independent [19], [26] [27]. Our results are in agreement with these findings, which indicate that GLP-1 does not play a major role in pregnancy-induced ß-cell proliferation.

Pregnant *Gcg^gfp/gfp^* mice showed higher blood glucose levels and lower insulin levels under *ad libitum* feeding than pregnant control mice in spite of comparable increases in ß-cell mass. Yet, as mentioned above, pregnant *Gcg^gfp/gfp^* mice showed lower insulin levels and higher glucose levels under *ad libitum* feeding. And these results suggested that pregnant *Gcg^gfp/gfp^* mice have a higher threshold for insulin secretion in response to a moderate elevation in blood glucose levels, and the absence of proglucagon-derived peptides, most likely GLP-1 is probably responsible for the altered threshold for insulin secretion. Nevertheless, after the oral administration of glucose, serum insulin levels were higher in pregnant *Gcg^gfp/gfp^* mice than in pregnant control mice, which indicated that insulin secretion in response to a rapid elevation in blood glucose is enhanced in *Gcg^gfp/gfp^* mice.

*Gcg^gfp/gfp^* mice showed a decreased pregnancy rate and smaller litter size as compared with control mice. In addition, a higher rate of embryo resorption and smaller litter size were observed in *Gcg^gfp/gfp^* females as compared with control females (Fig. 1). Given that the genotypes of the pups conformed with the expected Mendelian ratio, the resorption and/or growth failure of the fetuses were not affected by the fetal genotype. In *Gcgr^−/−^* mice, which also lose half of the fetuses during late gestation [20], it was demonstrated recently that expression of the genes for IGF-1, IGF-1 receptor and GLP-1 in the placenta was significantly down-regulated in *Gcgr^−/−^* mice [28]. As IGF-1 is a candidate for a regulator of placental nutrient transport [29], attenuated IGF-1 signaling is one of the possible causes of fetal loss caused by the absent action of glucagon. The cause of this partially defective fertility in *Gcg^gfp/gfp^* mice is obscure, however, chronic hypoinsulinemia and mild hyperglycemia during late gestational periods in *Gcg^gfp/gfp^* females are possible causes. Changes in other peptides/hormones that are involved in regulation of reproduction may be also involved [30]. It is also noteworthy that amino acid metabolism is altered in both *Gcg^gfp/gfp^* and *Gcgr^−/−^* mice and that both show hyperaminoacidemia [31], [32], as hyperaminoacidemia is observed in poorly controlled GDM and is associated with poor outcome [33].

Pups from *Gcg^gfp/gfp^* dams were lost more frequently within 36 hours after birth than those from control dams, and neonatal survival was not improved in multiparous *Gcg^gfp/gfp^* dams, in contrast to multiparous control dams. These results suggest that *Gcg^gfp/gfp^* dams have defects in relation to improving breeding capacity. Given that it has been shown that *Glp1r* null mice (*Glp1r^−/−^*) have defects in learning and memory [34], [35], the lack of GLP-1 might be responsible for the partially impaired breeding capacity of *Gcg^gfp/gfp^* dams.

In the present study, we analyzed fertility and pregnancy-associated change in pancreatic islet function in the absence of proglucagon-derived peptides. Our data clearly showed that proglucagon-derived peptides, including glucagon and GLP-1, are not required for reproduction and pregnancy-induced β-cell proliferation. Nevertheless, female *Gcg^gfp/gfp^* mice showed a decreased pregnancy rate, smaller litter size, insufficient insulin secretion during pregnancy and defects in breeding capacity. Multiple bioactive peptides, including glucagon and GLP-1, are produced from proglucagon and it is impossible to ablate one of these peptides selectively. Ablation of the glucagon receptor results in α-cell hyperplasia, markedly increased GLP-1 production and lower blood glucose levels. Future studies that employ multiple genetic models, such as *Gcg^gfp/gfp^* and *Gcgr^−/−^*, should further elucidate the role of proglucagon-derived peptides in pregnancy and reproduction.
